# Prevalence and patterns of post-traumatic stress disorder in victims of intimate partner violence

**DOI:** 10.1192/j.eurpsy.2024.1382

**Published:** 2024-08-27

**Authors:** F. Tabib, F. Guermazi, R. Masmoudi, D. Mnif, I. Feki, I. Baati, J. Masmoudi

**Affiliations:** Department of psychiatry A, UHC Hedi Chaker, Sfax, Tunisia

## Abstract

**Introduction:**

It’s a well-known fact that violence, particularly repetitive violence or violence lasting several years, as is often the case with intimate partner violence (IPV), has a severe psycho-traumatic impact. Although not all women are affected to the same degree or in the same way, post-traumatic stress disorder (PTSD) is the most common mental health consequence of IPV.

**Objectives:**

To assess the psycho-traumatic impact of IPV on female victims. To study the factors associated with PTSD among these women.

**Methods:**

We conducted a descriptive and analytical cross-sectional observational study, carried out over a 10-month period from March 2021 to December 2021, among female victims of IPV consulting psychiatric emergencies at UHC Hédi Chaker, Sfax, Tunisia for medical expertise at the request of the court. We studied the PTSD in these women using the Post Traumatic Stress Disorder Checklist Scale (PCLS).

**Results:**

The total number of participants was 120 with an average age of 37.27 years. The majority had secondary education or less (62.5%), were professionally active (53.3%), and were financially dependent on their partners (26.7%). As for the women’s clinical characteristics, 19.2% were under psychiatric care, 15% had attempted suicide and 10% had a history of childhood abuse. Regarding the couple’s profile, marriage was arranged in 58.3% of cases, and the average duration of marriage was 12.34 years, exceeding 10 years in 44.2% of cases.

The impact reported by our women was 100% psychological and 96.7% familial. As a result, 75.8% had sought help from family and friends, and 55.8% had decided to separate from their partners.

According to the PCLS scale, 78.3% of female victims showed PTSD with a positive score > 44. It was associated with a higher number of suicide attempts (p=0.04), a marriage duration exceeding 10 years (p=0.02), help-seeking (p=0.001), and divorce (p=0.014).

**Conclusions:**

PTSD is a particularly serious psychiatric condition. However, its impact remains insufficiently understood and taken into account in medical, psychological, social, and legal care. Knowing the psycho-traumatic consequences of violence is absolutely essential to better protect, support, and care for victims.

**Disclosure of Interest:**

None Declared

